# Underlying mechanisms of exercise protection against diabetes mellitus: a narrative review

**DOI:** 10.3389/fendo.2026.1854074

**Published:** 2026-07-23

**Authors:** Sizhe Wang, Fan Lu, Yuelin Liu, Ruyi Xu, Guannan Zhang, Yuxin Miao, Shanshan Lu, Zixu Liu, Weiqian Huang, Jinqi Meng, Danyang Tian

**Affiliations:** 1 Department of Physiology, Institute of Basic Medicine, Hebei Medical University, Shijiazhuang, China; 2 Department of Dermatology, The Second Hospital of Hebei Medical University, Shijiazhuang, China; 3 Hebei Institute of Sports Science, Key Laboratory of Training Load Diagnosis and Regulation for Elite Athletes, Shijiazhuang, China; 4 Department of Sports, Hebei Medical University, Shijiazhuang, China; 5 The Key Laboratory of Neural and Vascular Biology, Ministry of Education, Hebei Medical University, Shijiazhuang, China

**Keywords:** diabetic complications, exercise, insulin sensitivity, type 1 diabetes mellitus, type 2 diabetes mellitus

## Abstract

Diabetes mellitus is a significant metabolic disorder posing a serious threat to human health. While current pharmacological therapies are relatively well-established, they are often associated with certain side effects. As an economical and low-risk intervention, exercise has emerged as a pivotal non-pharmacological strategy in diabetes management. This narrative review summarizes the mechanisms by which exercise ameliorates both type 1 and type 2 diabetes, explores its beneficial impacts on diabetes-related complications, and compares the efficacy of different exercise modalities.

## 1 Introduction

Diabetes mellitus, a prevalent metabolic disease characterized by inadequate blood glucose control, ranks as a WHO top-ten cause of death and the sixth leading fatal noncommunicable disease globally (1). According to the International Diabetes Federation, approximately 589 million adults were living with diabetes in 2024, which equates to a population prevalence of roughly 11.1%, and the global adult diabetic population is predicted to reach an astonishing 853 million by 2050 (2). There were 3.4 million deaths caused by diabetes mellitus in 2024. Diabetes mellitus exerts its systemic damage primarily through chronic hyperglycemia-induced vascular and neural pathology. Complications are categorized into macrovascular diseases (accelerated atherosclerosis leading to coronary artery disease, stroke, and peripheral arterial disease) and microvascular diseases (diabetic retinopathy, nephropathy, and neuropathy). A critical clinical sequela is the diabetic foot, resulting from the interplay of neuropathy, ischemia, and infection, which significantly increases the risk of lower-extremity amputation. So diabetes has become a serious health issue threatening populations globally (2).

## 2 Main types and pathological characteristics of diabetes mellitus

The sources of blood glucose include: hepatic glucose output via glycogenolytic and gluconeogenic pathways, and exogenous carbohydrate absorption from enteral sources (3). The primary fates of blood glucose encompass oxidation for energy, glycogen synthesis for storage, and conversion to non-carbohydrate substrates like fats. Pathological excretion occurs only when hyperglycemia exceeds the renal threshold. The maintenance of blood glucose homeostasis is achieved through a complex hormonal regulatory system. This system relies on a delicate balance between the primary hypoglycemic hormone (insulin) and glycemic (including glucagon, epinephrine, cortisol, and growth hormone). Their antagonistic and synergistic interactions precisely coordinate hepatic glucose production, peripheral tissue glucose uptake and utilization to ensure stable glycemic concentrations (4, 5). Diabetes mellitus can be defined as a consequence of failures in the glucose regulatory system, such as β-cell dysfunction leading to reduced insulin output or the emergence of insulin resistance (IR).

Diabetes mellitus comprises several types, among which Type 1 and Type 2 are the most predominant subtypes.

### 2.1 Type 1 diabetes

T1DM is an autoimmune condition with the destruction of pancreatic islet β-cells by the immune system, leading to the deficiency of insulin and high blood glucose levels (6). This disease occurs in both classic juvenile-onset and adult-onset autoimmune diabetes variants. Patients with T1DM often develop concomitant autoimmune disorders, including autoimmune diseases, thyroid disease, celiac disease, autoimmune liver disease, vitiligo, collagen vascular diseases, and myasthenia gravis (7–9).

### 2.2 Type 2 diabetes

IR and pancreatic β-cell failure constitute a hallmark feature of T2DM (10). The predominant risk factors for T2DM include overweight/obesity, elevated blood pressure and glucose, aging, dietary patterns high in fat and sugar, a sedentary lifestyle, and urbanization (11). Among people with T2DM who are also overweight, approximately one-third are physically inactive (12). The accumulation of excessive ectopic fat promotes IR and triggers adverse metabolic alterations (13). Pancreatic β-cell dysfunction manifests early in the pathogenesis of both T1DM and T2DM (14). Critically, maternal hyperglycemia and gestational diabetes mellitus are associated with offspring precursors of T2DM, such as IR, further underscoring its significant impact on pancreatic β-cell development and function (15).

## 3 Current treatment approaches

The management of T1DM necessitates an integrated approach combining insulin replacement therapy, continuous glucose monitoring(CGM), medical nutrition therapy, and structured exercise interventions to achieve optimal glycemic control (16). The cornerstone of management remains intensive insulin replacement therapy, administered via multiple daily injections or insulin pumps, guided by continuous or frequent blood glucose monitoring. Adjunctive administration of non-insulin agents (e.g., pramlintide, SGLT2 inhibitors) has limited therapeutic effect (17). Frequent insulin administration leads to difficulty in persistence, including the inescapable patient burden of daily self-management, the persistent risk of iatrogenic hypoglycemia, the complexity of dose adjustment, and the considerable psychological impact, all of which can compromise glycemic control and quality of life (18). The management of T1DM primarily relies on conventional insulin therapy and adjunct pharmacotherapies. Sotagliflozin, as an adjunct to insulin therapy, has been shown to improve glycemic control in patients with T1DM. However, its use is associated with a significantly elevated risk of diabetic ketoacidosis, necessitating strict patient selection and careful clinical management (19). Current management of T1DM is fraught with limitations including significant treatment burden, persistent risk of glycemic excursions, and negative impacts on psychological well-being and quality of life. Furthermore, emerging regenerative approaches—including gene therapy, stem cell-based interventions—demonstrate considerable potential for future clinical applications (20). Despite significant progress in novel therapies, translating current research findings from bench to bedside will require considerable time.

Management of T2DM requires multifactorial intervention including health behaviors, glycemic targets for macrovascular and microvascular complications, glycemic monitoring, and pharmacotherapy tailored to individual phenotypes (21). The pharmacological management of T2DM follows a stepwise treatment approach. Metformin is established as the first-line therapeutic strategy for T2DM, owing to its well-documented efficacy and favorable cost-effectiveness profile (22). Metformin exerts its therapeutic effects by increasing insulin sensitivity and stimulating glucose uptake in skeletal muscle, while inhibiting both hepatic glucose production and intestinal glucose absorption. However, its use is frequently associated with significant gastrointestinal adverse effects (21). Sulfonylureas and glinides serve as second-line therapeutic agents for T2DM mellitus with demonstrated efficacy in glycemic management. Sulfonylureas and glinides exert their hypoglycemic effects through multiple mechanisms: primarily by stimulating insulin release from pancreatic β-cell, and additionally by suppressing glucagon secretion and enhancing tissue sensitivity to insulin (23). However, treatment-associated hypoglycemia, particularly resulting from sulfonylurea and insulin therapies, may confer adverse cardiovascular effects (24). If glycemic targets are not achieved, GLP-1 receptor agonists are preferentially recommended as add-on agents due to their potent glucose-lowering efficacy and associated weight loss benefits, rather than initiating insulin. Nevertheless, in cases of severe hyperglycemia, immediate insulin therapy is required (25). Advances in technology now allow for the use of smart insulin pumps, integrated with CGM, to achieve more precise glycemic control (26). Despite these advances in pharmacotherapy, achieving sustained glycemic control without adverse effects remains a challenge. In this context, physical exercise emerges as a powerful adjunctive strategy.

## 4 The benefits of exercise

Physical exercise plays a crucial role in both the prevention and management of multiple chronic conditions, including but not limited to cardiovascular disorders, Parkinson’s disease, and non-alcoholic steatohepatitis (27). Higher cardiovascular health scores, which include physical activity as a core component, are associated with reduced all-cause and cardiovascular mortality in obese adults, supporting the long-term prognostic value of exercise in metabolically high-risk populations. Regular physical exercise is associated with improved glycemic control, a reduced risk of diabetes-related complications, and enhanced cardiovascular health parameters in individuals with T1DM, without increasing the incidence of severe hypoglycemia requiring assistance (28). Studies have shown a superior therapeutic effect of exercise interventions over metformin administration in the management of prediabetes. Additionally, aerobic interval exercise and combined exercise emerged as the most efficacious modalities for improving glucose metabolism in both prediabetes and T2DM (29). Regular physical exercise demonstrably lowers glycated hemoglobin (HbA1c), reflecting better long-term glycemic management. The concurrent administration of structured exercise (moderate-to-vigorous aerobic physical activity, minimum 150 min/week of moderate-intensity or 75 min/week of vigorous-intensity or an equivalent combination of both) and liraglutide resulted in a greater reduction in the severity of metabolic syndrome, abdominal adiposity, and systemic inflammation compared to either intervention alone, suggesting a potentially superior effect on cardiometabolic risk mitigation (30).

## 5 Different types of physical exercise

Physical exercise is broadly categorized into aerobic and anaerobic forms based on the dominant metabolic pathways utilized, though most exercises involve an integration of multiple energy systems. Aerobic exercise—such as walking, cycling, jogging, and swimming—is characterized by rhythmic, sustained contractions of large muscle groups, primarily supported by oxidative metabolism. In contrast, resistance (or strength) training involves exercises performed with external loads (e.g., free weights, weight machines, elastic bands, or bodyweight), which predominantly utilize anaerobic pathways for energy production (31).

**Figure 1:**
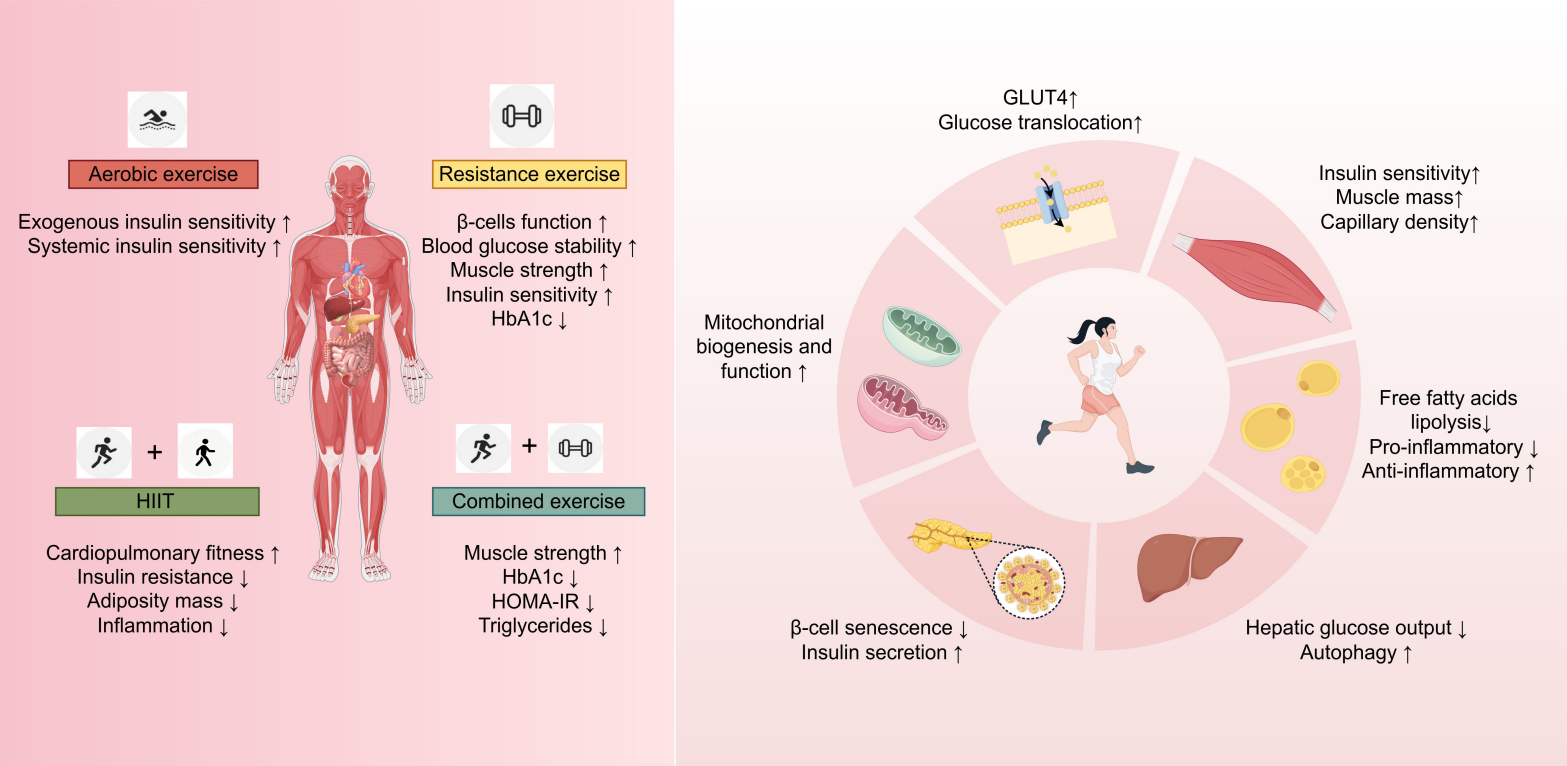
Comparative effects of aerobic, resistance, HIIT, and combined exercise on metabolic parameters in diabetes. The figure illustrates the distinct and shared effects of each exercise modality on glucose metabolism, insulin sensitivity, mitochondrial function, inflammatory markers, and other metabolic outcomes. ↑ indicates increase; ↓ indicates decrease. GLUT4, glucose transporter type 4; HbA1c, glycated hemoglobin; HIIT, high-intensity interval training; HOMA-IR, homeostatic model assessment for insulin resistance. Created with Figdraw.com.

Both single bouts and regular programs of prenatal exercise are associated with significant improvements in maternal glycemic control, with effect sizes being particularly pronounced in women with diabetes (32). Progressive aerobic or resistance exercise training, when implemented independently or in combination without dietary modifications, significantly ameliorates cardiometabolic risk factors (e.g., optimizing blood pressure and lipid profile), body composition (*e. g.*, reducing visceral and total fat), and glucose homeostasis (e.g., lowering HbA1c and enhancing insulin sensitivity) (33–38). A comparative overview of different exercise modalities is presented in **Figure 1**.

### 5.1 Aerobic exercise

Specifically, it is recommended that patients with T2DM engage in physical exercise for at least 150 minutes per week, achieving a target heart rate of 70% of maximum heart rate, through regular activities such as walking or jogging. Walking, in particular, has been demonstrated to improve glucose metabolism and cardiorespiratory fitness, thereby contributing to better health outcomes and quality of life in individuals with IR (39). Compared to walking, cycling and jogging are associated with greater energy expenditure, more pronounced reduction in blood glucose, and concomitantly higher risk of hypoglycemia (40). Swimming exercise alleviates T2DM by improving glucose intolerance and selectively mitigating endoplasmic reticulum stress in skeletal muscle (41).

#### 5.1.1 T1DM

Aerobic training for T1DM patients can enhance their sensitivity to exogenous insulin (42). In T1DM, aerobic training rapidly increases muscle activity and promotes non-insulin-mediated glucose utilization. However, its impact on HbA1c is modulated by the occurrence of hypoglycemia and nutritional intake (43). Aerobic exercise elicits the most pronounced glycemic reductions in adults with T1DM, surpassing both interval and resistance training (44). Prolonged moderate-intensity exercise (≥30 minutes duration) preferentially utilizes blood glucose as the primary metabolic substrate, thereby significantly increasing hypoglycemia risk in individuals with T1DM (45).

#### 5.1.2 T2DM

A finding identified miR-19b-3p as an aerobic exercise-induced miRNA that regulates skeletal muscle glucose metabolism. MiR-19b-3p coordinately improved skeletal muscle glucose metabolism by potentiating both the insulin signaling pathway (via Akt/AS160) and the contraction-mediated pathway, alongside an increase in mitochondrial protein abundance (46). However, aerobic exercise did not result in a statistically significant reduction in HbA1c (47). This may be explained by the small sample size. Short-term aerobic exercise enhances whole-body insulin sensitivity mainly through increasing peripheral insulin sensitivity, with no significant contribution from hepatic insulin sensitivity (48).

Aerobic exercise improved cardiac function in mice via activation of the parasympathetic nervous system, which mitigated oxidative stress (as evidenced by reduced lipid peroxidation) and promoted mitochondrial dynamic homeostasis (49).

### 5.2 Resistance exercise

Training with free weights, machines, or resistance bands for multiple sets (e.g., 2-4) of moderate repetitions (e.g., 8-12) at a challenging load are resistance exercise to enhance muscular strength, hypertrophy, and endurance. Resistance training serves as an effective non-pharmacological intervention to enhance cardiovascular fitness, augment bone mineral density, and increase muscle mass (50–52). Preservation of muscle mass and strength reduces the risk of multiple diabetes-related comorbidities, such as microvascular and macrovascular complications, and osteoporosis, while also offering protective effects against obesity. Resistance Exercise enhances skeletal muscle functional capacity through dual mechanisms: improved contractile properties and augmented metabolic function (53). Resistance training represents an essential component of the recommended weekly physical exercise for individuals with T1DM and T2DM (54, 55).

#### 5.2.1 T1DM

Through the induction of circulating humoral factors, resistance training upregulates GLUT2 and glucokinase expression, suppresses apoptotic pathways, thereby protecting pancreatic β-cells from inflammatory injury, increasing β-cell mass, and improving glycemic control in streptozotocin (STZ)-induced T1DM animal models (56). Studies have demonstrated that despite the superior acute glucose-lowering capacity of aerobic exercise, resistance exercise significantly increases 24-hour time in range, suggesting a potentially greater clinical value of resistance exercise for daily glycemic control in individuals with T1DM (57). In T1DM patients already engaged in regular aerobic exercise, the addition of resistance training did not further reduce HbA1c. However, despite no significant change in body weight, strength training significantly improved waist circumference, reflecting a favorable body composition remodeling (58). A sex-specific difference was observed among patients with T1DM, with females exhibiting more stable glycemic control during resistance exercise sessions compared to males (59). Performing resistance exercise prior to HIIT represented a beneficial strategy for reducing hypoglycemia risk in individuals with T1DM (60).

#### 5.2.2 T2DM

In T2DM patients, strength training produced significant improvements in body composition—reducing both BMI and body fat percentage—without a concomitant significant reduction in total body weight (61). The intervention of progressive sandbag exercise elicited significant enhancements in glycemic control and muscular strength among T2DM mellitus patients complicated with sarcopenia (62). Resistance training demonstrated a glucose-lowering effect comparable to aerobic training in patients with T2DM, and this effect is positively correlated with improvements in muscle strength (63). In normal-BMI individuals with T2DM, strength exercise indicated greater efficacy in lowering HbA1c compared to isolated aerobic training, whereas combined aerobic and strength training showed comparable glycemic benefits to strength training alone (47).

In T2DM, resistance exercise improves HbA1c by enhancing insulin-mediated glucose disposal and expanding the skeletal muscle glucose sink. In T1DM, despite increases in strength and reductions in daily insulin requirements, resistance exercise typically fails to produce robust HbA1c reductions due to the underlying absolute insulin deficiency (47). Of note, resistance exercise plays distinct roles in T1DM and T2DM. In T1DM, where glycemic variability and hypoglycemia risk are the major concerns, resistance exercise primarily improves 24-hour time in range and glycemic stability. In T2DM, by contrast, its metabolic benefits are mainly mediated through enhanced muscle strength, improved insulin sensitivity, and reduced HbA1c.

### 5.3 HIIT

HIIT, a time-efficient exercise strategy characterized by brief bursts (10 seconds to 5 minutes) of near-maximal exertion interspersed with active recovery periods, including cycling, rowing, or running protocol (64). HIIT exerts a positive role in diabetes and its complications management by reducing short-term glycemic variability and improving cardiorespiratory fitness (65, 66).

#### 5.3.1 T1DM

For individuals with T1DM, HIIT produces improvements in cardiorespiratory fitness and short-term glucose levels, without affecting long-term measures of glycemic control including HbA1c and fasting glucose (67). The 12-week HIIT intervention did not elicit a significant reduction in HbA1c of T1DM. However, subanalysis indicated that higher intervention adherence is associated with favorable glycemic outcomes (68). During HIIT sessions, some participants reported hypoglycemia-like symptoms, such as dizziness, despite a measured upward trend in blood glucose levels (69). Importantly, this acute hyperglycemic response during HIIT does not negate the risk of late-onset hypoglycemia occurring hours after exercise, which can be mitigated by reducing the evening basal insulin dose (70). These findings underscore the critical importance of either real-time glycemic monitoring or personalized exercise programming to maintain patient safety during physical exercise. In obese patients with T1DM, HIIT alone do not result in significant weight loss (68).

#### 5.3.2 T2DM

In T2DM patients, HIIT significantly improves insulin resistance—outperforming continuous aerobic training—and produces modest weight loss. Fasting glucose reductions are pronounced in those with or at risk for T2DM, while HbA1c shows a statistically significant but modest decrease (71). Although HIIT does not appear to alter glycemic control in obese individuals with T2DM, it produces clinically relevant reductions in adiposity and hepatic steatosis, in addition to its superior ability to preserve lean mass compared to conventional moderate-intensity exercise (65). HIIT ameliorates hepatic steatosis, inflammation, and insulin resistance via orchestrating a shift in hepatic macrophages towards the M2 phenotype and enhancing mitochondrial function, leading to improved insulin signaling (IRS1/PI3K/AKT), promoted fatty acid oxidation, suppressed lipogenesis, and reduced inflammation (72).

The lack of a statistically significant change in HbA1c among T1DM patients after exercise might be explained by increased caloric intake, reduced insulin dosing during exercise, insufficient energy availability, and the limited sensitivity of HbA1c to glycemic variability in T1DM (73). The discrepancies in body composition may be attributed to dietary factors. In T2DM, weight loss results from improved insulin resistance. In T1DM, however, exogenous insulin-induced lipolysis and hypoglycemia-driven carbohydrate intake counterbalance energy loss, leading to weight stability (68).

### 5.4 Combined exercise

Combined exercise training refers to a structured exercise regimen that integrates two or more distinct modes of exercise, most commonly aerobic (endurance) training and resistance (strength) training, within a single intervention program.

#### 5.4.1 T1DM

Resistance and combined exercise demonstrate superior efficacy to aerobic exercise in improving muscle strength in individuals with T1DM (74). Beyond improvements in muscle strength, a recent network meta-analysis demonstrated that multi-component exercise (combined aerobic and resistance training) was the only exercise modality that significantly reduced HbA1c in T1DM patients, with the highest probability of being the most effective intervention. In contrast, aerobic exercise, resistance training alone, and high-intensity interval training failed to achieve statistically significant reductions (75). Furthermore, a dose-response analysis revealed that even a modest dose of combined exercise—well below the minimum weekly physical activity recommended by the American Diabetes Association—was sufficient to achieve the minimal clinically important difference in HbA1c reduction (75). These findings suggest that combined aerobic and resistance training, rather than single-modality exercise, should be prioritized for glycemic management in individuals with T1DM.

#### 5.4.2 T2DM

Combined exercise in T2DM can reduce HOMA-IR, suggesting an improvement in insulin sensitivity (76). However, some RCTs have reported that although HOMA-IR decreased, it did not reach a significant level (77).

The optimal 60:40 aerobic-to-resistance training ratio synergistically lowers HbA1c and reduces microvascular complication risk through complementary mechanisms. In light of substantial evidence supporting the role of exercise in the treatment and management of T2DM, the American College of Sports Medicine has established structured exercise protocols tailored for individuals at high risk of IR (78). Among adults with overweight or obesity, aerobic exercise—either alone or in combination with resistance training—is associated with a significant improvement in the composite CVD risk profile. Resistance exercise alone failed to demonstrate a statistically significant benefit over the control condition (79).

Although the combined aerobic and resistance exercise intervention elicited more HbA1c declining effects compared to single-modality training, this approach was limited by its extended total training duration relative to aerobic or resistance training alone (80). Compared to single-modality training, combined exercise provided superior glycemic control. For the management of exercise-induced lipid improvements, supervised resistance training demonstrates superior efficacy in modulating cholesterol profiles (TC, LDL, and HDL), whereas combined exercise yields the most pronounced reductions in triglycerides (81). Consequently, exercise prescriptions should be individualized based on the specific dyslipidemia phenotype.

For patients with T1DM, combined exercise should be prioritized to improve both HbA1c and muscle strength, with even modest doses conferring clinical benefits. For T2DM patients, an aerobic-to-resistance training ratio of 60:40 is recommended; however, exercise prescriptions should be individualized based on the patient’s lipid phenotype, with careful consideration of the trade-off between time commitment and metabolic benefits.

## 6 The physiological mechanisms of exercise in improving glucose metabolism

### 6.1 T1DM

**Figure 2:**
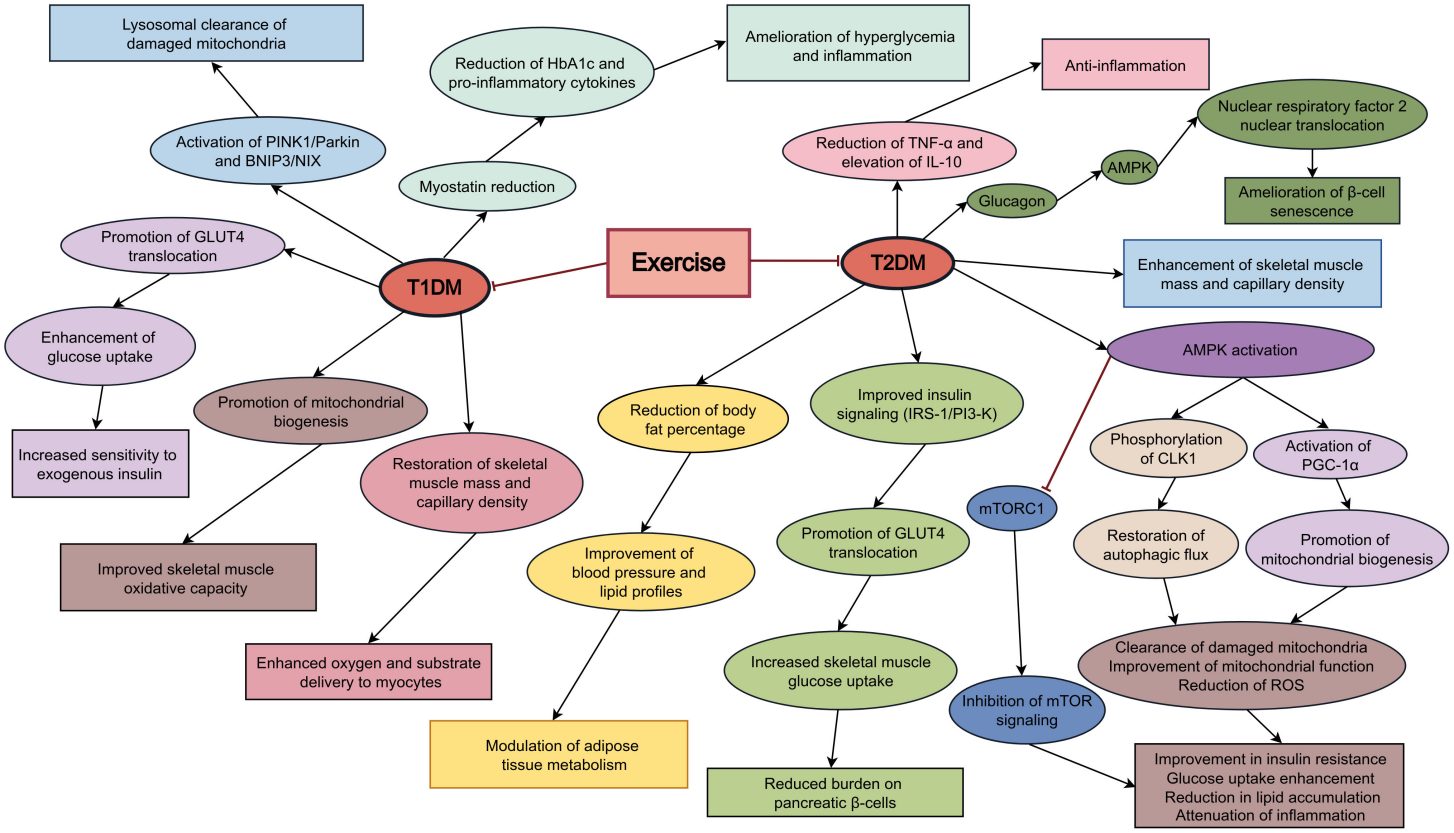
Exercise-induced protective mechanisms in T1DM and T2DM. The figure compares the distinct molecular pathways through which exercise exerts protective effects in type 1 diabetes (mitochondrial quality control, GLUT4 translocation, exogenous insulin sensitivity) and type 2 diabetes (adipose tissue metabolism, IRS-1/PI3-K signaling, β-cell protection). Abbreviations as defined in the text. Created with Figdraw.com.

The variability in glycemic responses to physical exercise among individuals with T1DM arises from a complex interplay of multiple determinants. These encompass both exercise-related parameters (modality, intensity, and duration) and intrinsic or extrinsic factors, including and not limited to: pre-exercise plasma glucose levels, individual insulin sensitivity profiles, residual β-cell function, fitness status, circadian timing of activity, nutritional intake (both timing and composition), insulin administration protocols (dosage and timing), psychological stress levels, sleep quality preceding exercise, and biological sex differences (31, 82). Beyond its established metabolic benefits, exercise plays a particularly critical role in breaking the vicious cycle of weight gain and escalating insulin dependence observed in individuals with T1DM who develop obesity—a cycle from which patients struggle to break free without effective intervention (83). The protective mechanisms of exercise against diabetes are illustrated in **Figure 2**.

#### 6.1.1 Exercise enhances insulin sensitivity and non-insulin-mediated glucose uptake

In T1DM adults, higher objectively measured daily physical activity (step count and MVPA time) correlates with better long-term glycemic control, lower insulin needs, and greater insulin sensitivity (84). A key molecular mechanism by which exercise improves glucose disposal in T1DM is the upregulation of glucose transporter type 4 (GLUT4) in skeletal muscle. In a T1DM rat model, six weeks of exercise training—whether resistance, low-intensity aerobic, or high-intensity aerobic—induced tissue-specific elevations in GLUT4 content in both oxidative and glycolytic portions of the vastus lateralis and gastrocnemius muscles (85). This exercise-induced GLUT4 upregulation was concomitant with improved glucose tolerance and reduced exogenous insulin requirements, indicating that enhanced GLUT4 expression in skeletal muscle augments non-insulin-mediated glucose uptake and partially compensates for the insulin-deficient state in T1DM (85). These findings provide direct mechanistic evidence linking exercise training to improved glucose handling in T1DM through skeletal muscle GLUT4 content elevation.

#### 6.1.2 Exercise increases skeletal muscle mass and capillary density

Muscle atrophy and weakness are associated with T1DM (86). An intervention comprising high-intensity exercise and concomitant insulin therapy over a two-week period led to a significant restoration of muscle mass and strength (87). Patients with T1DM also exhibit a significantly lower skeletal muscle capillary density compared to healthy individuals. In contrast to this deficit, a regimen of swimming exercise served as an effective non-pharmacological intervention to augment capillary density (88). This angiogenesis enhances oxygen and substrate delivery to myocytes, thereby improving the metabolic capacity of skeletal muscle for glucose disposal.

#### 6.1.3 Exercise-driven metabolic enhancement via mitochondrial biogenesis and autophagy

Mitochondria are central organelles in cellular energy metabolism, responsible for ATP generation in all tissues. Due to their involvement in oxidative phosphorylation and substrate oxidation, mitochondria represent a major source of ROS production (89).

Skeletal muscle mitochondrial dysfunction may be mediated by elevated levels of reactive oxygen species (ROS), which are secondary to insulin deficiency, hyperlipidaemia and other metabolic abnormalities in T1DM (90). Non-obese adolescents with T1DM exhibited concomitant IR and skeletal muscle mitochondrial dysfunction. These two conditions are significantly associated, independent of glycemic control (HbA1c) (91). This finding suggests that mitochondrial dysfunction is not merely secondary to hyperglycemia, but may represent an intrinsic defect requiring targeted interventions—such as exercise—beyond conventional glucose-lowering therapy.

Mitophagy controls mitochondrial population by targeting damaged and depolarized mitochondria for degradation and recycling, a process essential for sustaining cellular energy metabolism (92). Young, active T1DM individuals exhibited skeletal muscle mitochondrial dysfunction—evidenced by reduced oxidative capacity (Complex II), elevated H_2_O_2_ emission (Complex III), and decreased calcium retention—alongside an accumulation of autophagic remnants. This occurred despite normal levels of key autophagic regulators, suggesting a defect in the execution rather than the signaling of autophagy (89).

The mechanism by which exercise counteracts muscle atrophy involves a precision enhancement of mitophagy. Exercise initiates a coordinated molecular response through pathways such as PINK1/Parkin and BNIP3/NIX, promoting the targeted sequestration and lysosomal degradation of damaged mitochondria (87). By clearing dysfunctional mitochondria and promoting biogenesis of new, efficient ones, exercise enhances skeletal muscle oxidative capacity and insulin sensitivity.

#### 6.1.4 Exercise ameliorates hyperglycemia and inflammation in T1DM

Myostatin, also known as growth/differentiation factor-8, functions as a potent inhibitor of skeletal muscle growth across multiple animal species, including humans (93). Elevated serum myostatin levels have been observed both in children with T1DM and in STZ-induced T1DM mouse models (94, 95). Aerobic exercise lowers myostatin levels in plasma and skeletal muscle of T1DM rats, and this reduction is positively correlated with both HbA1c improvement and decreased pro-inflammatory cytokines (IL-6, TNF-α, IL-1β). Exercise-induced myostatin inhibition plays a causal role in the amelioration of hyperglycemia and inflammation in T1DM (96).

### 6.2 T2DM

#### 6.2.1 Exercise ameliorates β-cell senescence

Endurance exercise-induced elevation of serum glucagon activates AMP-activated protein kinase(AMPK) signaling in pancreatic β-cells, promoting nuclear respiratory factor 2 nuclear translocation, which reduces the expression of senescence markers and ultimately ameliorates β-cell senescence, offering therapeutic potential for T2DM (97). Preservation of β-cell health is of clinical significance, as progressive β-cell dysfunction and loss constitute a hallmark of T2DM pathogenesis. By attenuating β-cell senescence, exercise may preserve endogenous insulin secretory capacity, thereby slowing disease progression and potentially delaying the transition to insulin therapy.

mTORC1 coordinates anabolic and catabolic metabolism by modulating key transcription factors (98). Parallel to AMPK-mediated anti-senescence, nutrient signaling via the mTORC1-PDX1 axis also regulates β-cell function. Amino acids activate mTORC1, which phosphorylates PDX1 at serine 61, enhancing its stability and transcriptional activity to promote insulin expression and β-cell proliferation. Under chronic nutrient excess, sustained PDX1 phosphorylation drives pathological hyperinsulinemia, obesity, and hepatic steatosis (99). Exercise, via AMPK activation, inhibits mTORC1 and may temper excessive PDX1 phosphorylation, complementing its anti-senescence effects to preserve β-cell health.

#### 6.2.2 Exercise enhances insulin sensitivity and lowers blood glucose

Skeletal muscle, being the major tissue for glucose disposal stimulated by insulin, develops IR that constitutes a core mechanism underlying the pathogenesis of T2DM (100). During short-term, high-intensity exercise, muscle glycogen serves as the predominant fuel source, whereas blood glucose becomes increasingly important during prolonged exercise, contributing up to 40% of total oxidative metabolism when glycogen stores are depleted (101–104).

Exercise training ameliorates IR probably through synergistic enhancement of glucose metabolism, insulin signaling, and muscle lipid oxidation (105). Skeletal muscle accounts for roughly 85% of insulin-dependent glucose uptake, demonstrating a 50-fold augmentation during intense exercise (106). Insulin-responsive glucose uptake in skeletal muscle is principally mediated by GLUT4 (107). Muscle contraction during endurance exercise training mediates GLUT4 translocation to the plasma membrane and t-tubules, significantly enhancing glucose uptake in working muscle. Early-phase endurance training (1 week) increases GLUT4 protein via posttranslational mechanisms without changes in GLUT4 mRNA. The subsequent rise in GLUT4 mRNA with prolonged training results from the cumulative effect of repeated transient transcriptional activation (~1.8-fold) occurring 3 hours after each exercise bout (108). In obese individuals with T2DM, short-term high-intensity aerobic training (7 days) enhanced whole-body insulin-mediated glucose disposal via increased skeletal muscle GLUT4 protein content, independent of any alterations in the canonical insulin signaling cascade (IRS1, IRS2, PI3K, Akt, and AS160) (109).

Muscle contraction during exercise elevates intracellular calcium, nitric oxide, ROS, and AMP/ADP ratios, which serve as metabolic stressors to activate key signaling kinases, including mitogen-activated protein kinases (MAPKs: ERK, p38, JNK), Ca²^+^/calmodulin-dependent kinase kinase, and AMPK (110). Activation of these signaling pathways enhances cellular uptake and oxidative metabolism of both glucose and fatty acids. Further research suggests that physical training improves hepatic glucose control and modifies whole-body lipid processing. One proposed mechanism involves the augmented activation of Insulin Receptor Substrate 1 (IRS-1), which in turn stimulates the Phosphatidylinositol 3-Kinase (PI3-K) pathway. This enhanced signaling promotes insulin sensitivity independent of changes in the abundance of relevant proteins (111). Concurrently, post-exercise elevation of muscle insulin sensitivity potentiates glycogen store replenishment (112). The enhancement of insulin sensitivity in skeletal muscle directly translates to improved whole-body glycemic control. This adaptation reduces the insulin demand on pancreatic β-cells, alleviating metabolic stress and breaking the vicious cycle of insulin resistance and β-cell decompensation characteristic of T2DM.

#### 6.2.3 Exercise increases skeletal muscle mass and capillary density

Patients with T2DM with normal body weight demonstrate a significant correlation with sarcopenia, along with generalized and progressive loss of skeletal muscle mass (113). Engaging in regular physical exercise is associated with improvements in muscular strength and endothelial function (51, 114).

In sedentary populations, robust evidence indicates that both moderate-intensity continuous training and high-intensity interval training (HIIT) are effective stimuli for inducing skeletal muscle capillarization, whereas low-intensity training regimens elicit markedly diminished adaptations (115). Increased skeletal muscle capillary density following AT is a key mechanism by which insulin sensitivity is improved in sedentary older adults at risk of T2DM (116). Increased capillary density expands the endothelial surface area available for insulin and glucose delivery to myocytes, while greater muscle mass provides a larger sink for glucose disposal. Together, these structural adaptations create a peripheral environment more conducive to maintaining glycemic homeostasis, counteracting the sarcopenia and microvascular rarefaction commonly observed in T2DM.

#### 6.2.4 Exercise enhances mitochondrial biogenesis and function

Endurance exercise training promotes cardiac glucose metabolic adaptation via an eNOS-dependent pathway that enhances mitochondrial biogenesis through the PGC-1α-mediated upregulation of key transcriptional regulators, including nuclear respiratory factor 1 and mitochondrial transcription factor A (117). Compared to moderate aerobic exercise, HIIT is a markedly more potent intervention for robustly enhancing mitochondrial function (including oxidative capacity, protein synthesis, and content) and improving insulin sensitivity in the older adult population (118). In obese, insulin-resistant women with polycystic ovary syndrome, a 12-week aerobic exercise intervention effectively restored skeletal muscle mitochondrial physiology—characterized by enhanced bioenergetic efficiency and reduced oxidative stress—to a level comparable to that of lean, insulin-sensitive controls. This amelioration of mitochondrial dysfunction is paralleled by a concomitant improvement in systemic insulin sensitivity (119). Improved mitochondrial function enhances the efficiency of substrate oxidation and reduces intramyocellular lipid accumulation, a key driver of insulin resistance.

#### 6.2.5 Exercise enhances metabolism by promoting autophagy

As a pivotal cellular homeostatic mechanism, autophagy plays an indispensable role in key metabolic tissues—including liver, muscle, and adipose tissue—as well as in pancreatic β-cell (120). Conversely, a dysfunction in the autophagic process is a well-established lesion critically implicated in the pathogenesis of T2DM and its associated complications (121). It is well-established that autophagy is compromised in T2DM, with notable downregulation of key autophagy genes in both the liver and skeletal muscle, leading to diminished autophagic flux. Physical exercise serves as a potent inducer of autophagic flux in key metabolic organs, including the liver, pancreas, and adipose tissue (122). Autophagy impairment, driven by mTORC1 hyperactivation and AMPK/SIRT1 insufficiency, underlies multi-organ metabolic dysfunction in T2DM, including adipose inflammation, hepatic steatosis, β-cell failure, muscle atrophy, and intestinal barrier disruption. Notably, exercise—through AMPK activation—effectively restores autophagic flux, positioning physical activity as a cornerstone non-pharmacological strategy to counteract autophagy-dependent metabolic deterioration in T2DM (123).

Exercise induces skeletal muscle to secrete Fibronectin 1, which activates hepatic autophagy and improves systemic insulin sensitivity via binding to the α5β1 integrin receptor in the liver (124). Exercise, as an effective intervention, has been shown to activate the BCL2-dependent autophagic pathway. This activation serves to restore and optimize intracellular conditions, thereby ultimately manifesting as improved glucose metabolism and overall metabolic health (122). A decline in skeletal muscle autophagic flux contributes to muscle atrophy, while its enhancement is beneficial for the maintenance of muscle mass (125). Exercise-elicited metabolic benefits mediated by AMPK/Sestrins-dependent autophagy and improved glucose metabolism in skeletal muscle (126). Furthermore,the upregulation of short-chain fatty acids induced by exercise may ameliorate IR. It is proposed that this beneficial effect is mediated through the activation of GPR43 receptors, which subsequently enhances autophagy in skeletal muscle cells (127). By restoring autophagic flux in metabolic tissues, exercise facilitates the clearance of protein aggregates and damaged organelles, maintains cellular homeostasis under metabolic stress, and improves insulin signaling.

#### 6.2.6 Exercise improves adipose tissue metabolism

Obesity represents a significant risk factor for the development of diabetes (128). Importantly, weight reduction accomplished by combined exercise and dietary interventions has been shown to achieve T2DM remission in nearly 80% of obese individuals (129). Endocrine dysfunction of adipose tissue in obesity plays a critical role in the development of insulin resistance. Obesity reduces spexin expression in adipose tissue, which in turn promotes insulin resistance in both adipose tissue and skeletal muscle. Exercise upregulates adipose tissue spexin expression, thereby ameliorating insulin resistance and alleviating T2DM (130). Adipose tissue may represent an initial site of IR, with its dysfunction potentially driving the subsequent development of whole-body IR in muscle and liver (131). Sedentary behavior is a contributor to IR in both skeletal muscle and adipose tissue, which manifest as the failure to adequately suppress lipolysis. The consequent rise in circulating free fatty acids drives ectopic deposition in non-adipose tissues, thereby perpetuating systemic metabolic dysfunction (132). Reducing sedentary behavior and increasing physical exercise can decrease body fat percentage, resulting in improved blood pressure and lipid profiles, which may subsequently reduce the risk of cardiovascular disease(CVD) (133). Subcutaneous abdominal adipose tissue (SAT), recognized as the largest adipose depot, serves as the primary source of free fatty acids delivered to the liver. Interventions including aerobic training, resistance training, and combined exercise training have been demonstrated to induce significant reductions in SAT. Among these, aerobic training appears to exert superior efficacy in reducing SAT volume (134).

The benefits of exercise training on metabolic attribute to a depot-specific adaptation, wherein visceral adipose tissue demonstrates markedly improvement in insulin sensitivity compared to its subcutaneous counterpart (135). Exercise can break the lipotoxicity cycle that perpetuates insulin resistance and glycemic deterioration in T2DM.

#### 6.2.7 Exercise modulates inflammatory factors

Upregulated TNF-α and downregulated IL-10 were observed in T2DM patients compared to healthy controls. The 8-week high-intensity interval training intervention elicited a favorable shift in the inflammatory profile of T2DM, characterized by reduced TNF-α and elevated IL-10 concentrations, suggesting an exercise-induced anti-inflammatory effect (72).

## 7 Exercise ameliorates diabetic complications

Diabetic complications arise from chronic hyperglycemia, which induces microvascular damage (leading to retinopathy, nephropathy, neuropathy) and accelerated macrovascular injury (predisposing to CVD). Exercise contributes to delaying the progression of chronic complications through improved glycemic control. Regular physical exercise, as a component of healthy lifestyle, is associated with a reduced risk of developing various complications in individuals with T2DM (136, 137). Physical exercise ameliorates CVD by enhancing mitochondrial homeostasis, through processes such as improved mitochondrial biogenesis and mitophagy (138).

The combined moderate intensity training and resistance training intervention elicited significant improvements in both body composition and cardiorespiratory fitness following the one-year trial (139). Evidence indicates that regular exercise training, particularly aerobic exercise, enhances cardiovascular and pulmonary function, as well as circulating glucose levels (140). To safely and effectively translate these physiological benefits into personalized exercise prescriptions for diabetic patients, advances in self-powered wearable devices now enable real-time monitoring of cardiovascular parameters during physical activity (141).

### 7.1 Exercise attenuates diabetic nephropathy

Diabetic Nephropathy disease progresses due to hyperglycemia-induced metabolic, hemodynamic, and inflammatory pathways, ultimately leading to renal fibrosis and functional decline. The pathogenesis of diabetic nephropathy involves the dysregulation of numerous miRNAs, which promotes pro-inflammatory and pro-fibrotic pathways (142). MicroRNAs play a key regulatory role in the pathogenesis of CVD and diabetes-related complications (143). In the arterial tissue of diabetic subjects, miR-181b expression is downregulated, contributing to endothelial dysfunction, vascular inflammation, and oxidative stress (144). Aerobic exercise ameliorates renal tubular interstitial fibrosis in high-fat diet/STZ-induced diabetic mice through AMPK activation-mediated promotion of mitochondrial autophagy in the kidneys (145, 146). Chronic exercise exerts beneficial effects on diabetic endothelial dysfunction, likely mediated through the activation of the AMPK signaling pathway. This activation leads to the upregulation of the downstream target miR-181b (144). Exercise-induced upregulation of miR-181b attenuates phosphatase and tensin homolog-mediated suppression of the PI3K/AKT pathway, ultimately ameliorating vascular inflammation, apoptosis, and renal injury in diabetic nephropathy (147).

### 7.2 Exercise alleviates diabetic neuropathy

Metabolic stress or microvascular ischemia lead to neuronal dysfunction and damage. Exercise significantly improves outcomes in diabetic peripheral neuropathy (DPN), as demonstrated by meta-analysis of 27 randomized trials. Combined endurance and sensorimotor training yielded the greatest benefits, enhancing static balance, functional mobility, nerve conduction velocity, and glycemic control (HbA1c). Based on strong evidence, this combined exercise modality is recommended for clinical management of DPN (148).

## 8 Conclusion

In conclusion, physical exercise serves as a highly beneficial and low-risk adjunctive strategy for both major forms of diabetes. Its pleiotropic benefits for glycemic regulation are achieved through pathways ranging from whole-body musculature enhancement to subcellular quality control, including increased muscle mass/strength/capillarization, improved anti-inflammatory milieu (e.g., IL-10/TNF-α ratio), and upregulated mitochondrial autophagy. Furthermore, exercise addresses key pathophysiological defects in T2DM by boosting insulin sensitivity and secretion. Exercise exerts beneficial effects through specific mechanisms that ameliorate diabetic complications. The importance of physical exercise in diabetes management warrants greater emphasis in standard clinical practice.
